# Student assistantship programme: an evaluation of impact on readiness to transit from medical student to junior doctor

**DOI:** 10.1186/s12909-022-03159-3

**Published:** 2022-02-14

**Authors:** Aloysius Chow, Shiwei Chen, Lucy Rosby, Naomi Low-Beer, Vishalkumar Girishchandra Shelat, Jennifer Cleland, Bernadette Bartlam, Helen Elizabeth Smith

**Affiliations:** 1 Lee Kong Chian School of Medicine, Nanyang Technological University Singapore, Nanyang, Singapore; 2 Tan Tock Seng Hospital, Tan Tock Seng, Singapore; 3 School of Primary, Community and Social Care, Faculty of Medicine and Health Sciences, Keele University, Keele, UK

**Keywords:** Transitions to practice, Preparedness, Undergraduate medical education, Junior doctors, Clinical supervisors, Evaluation, Workplace learning

## Abstract

**Background:**

Studies report that medical graduates are not prepared for practice as expected, and interventions have been developed to prepare them for practice. One such intervention is the assistantship, which provides hands-on opportunities to hone clinical skills and undertake responsibilities under supervision. The Lee Kong Chian School of Medicine (LKCMed) is Singapore’s newest medical school, and students undergo a Student Assistantship Programme (SAP) to prepare for practice as junior doctors (PGY1). This study evaluated the SAP from the students’ and clinical supervisors’ perspectives.

**Methods:**

Students completed online questionnaires to assess readiness for practice before and after SAP, and a subsample were interviewed about their experiences of SAP and its impact on their preparedness for PGY1. In addition, after our graduates had begun work as PGY1 doctors, their clinical supervisors completed an online questionnaire and were interviewed about the perceived benefits of SAP and the attributes of our graduates as junior doctors.

**Results:**

Fifty (96%) students completed the pre-SAP questionnaire and 46 (92%) completed the post-SAP questionnaire. Levels of preparedness increased post-SAP (mean scores range pre-SAP: 2.38 to 4.32 vs post-SAP: 3.08 to 4.48); so did opportunities to undertake PGY1 duties (pre-SAP: 56% vs post-SAP: 96%), and hands-on experience in medical emergencies (pre-SAP: 76% vs post-SAP: 89%).Experience of acute care situations increased except “paracetamol overdose”. Readiness to be first respondents in ten acute situations improved (statistically significant for asthma, chronic obstructive pulmonary disease exacerbation, gastrointestinal bleed, sepsis, and adverse drug reactions). Three themes emerged from twenty-five student interviews: learning about the work environment, opportunities to learn in a safe environment, and enhancing SAP for future students. Thirty-three supervisors completed the questionnaire, and 70% rated SAP positively in preparing students for PGY1. Eight supervisors interviewed shared positively about the content, timing, and duration of SAP; and suggested future SAPs help students to develop coping and reflective skills.

**Conclusions:**

The SAP improved students’ preparedness and experience across clinical areas, and students felt the SAP helped bridge undergraduate curriculum and work, provided opportunities to hone their skills and learn from junior doctors. Most clinical supervisors rated the SAP effective in preparing students for PGY1. This is the first formal evaluation of an assistantship in Singapore, and the findings are encouraging from the perspective of students and PGY1 supervisors.

## Background

It has been argued that junior doctors can never be fully prepared, due to the near-impossible possibility of being able to encounter every clinical scenario before starting work [1]. The perceived level of preparedness has been assessed in several settings internationally [2–4] and a recent systematic review of 87 articles of medical graduates’ preparedness for practice in the UK [5] concluded that generally medical graduates were not prepared for practice. The areas where graduates frequently reported under-preparedness related to prescribing, clinical reasoning and diagnoses, early management of emergency situations, wound suturing, central venous line and chest drain insertion, safety and error reporting, ethical and legal matters, and understanding the workings of clinical environments.

Unfortunately, unpreparedness impacts adversely on patient outcomes. The term Black Wednesday has been used in the UK to describe the first Wednesday of August, when there is a nationwide changeover of junior doctors in all hospitals. A study analysing 300,000 emergency admissions between 2000 to 2008 found a statistically significant increase of 6% to 8% in the odds of a patients dying on Black Wednesday compared to on Wednesday in the previous week [6]. In the USA, a higher mortality rate has been reported during annual turnover of junior doctors [7], although a more recent study did not find significant changes in mortality, but the lengths of patient stays were extended [8].

The interventions that have been developed to better prepare medical students for their roles as junior doctors can be categorised into three types: assistantships, induction, and shadowing [9]. Assistantships provide medical students with hands-on opportunities to hone their clinical skills as part of a clinical team, where they undertake defined responsibilities under appropriate supervision. Induction is when a medical graduate is familiarised to the new work environment and employment policies by the respective departments of the hospital. Shadowing refers to a short period allocated to new junior doctors, with the specific aim of building relationships with their supervisors and colleagues. Our understanding of the efficacy of each of these different approaches remains weak. A recent systematic rapid review of transition interventions indicated the evidence for assistantships was particularly sparce [5], and we have been unable to find any studies from an Asian setting. Existing evidence of the benefits assistantships is often subjective, been derived solely from self-reported student data [10–12]. This methodological weakness can be reduced by including the perspective of others in the clinical team when evaluating transition interventions on readiness to practice.

The Lee Kong Chian School of Medicine (LKCMedicine) is Singapore’s newest medical school, developed as a collaboration between Nanyang Technological University Singapore, and Imperial College London. The first cohort of MBBS students were admitted in 2013. Distinctive features of the MBBS programme include team-based learning, early patient contact, an extensively digitalised curriculum, a strong focus on professionalism and clinical communication throughout the five-year programme, and a Student Assistantship Programme (SAP) conducted after the final MBBS examination to prepare students for their transition to Postgraduate Year 1 (PGY1). During the SAP students undertake a series of summative workplace-based assessments (WBAs). Graduation is dependent on undertaking the SAP and completing the required WBAs to a satisfactory standard. The SAP provides opportunities for graduates to undertake under supervision the professional duties of a PGY1 doctor. Graduates take responsibility clerking patients, ensuring handovers are safe, managing acute care cases, assisting in procedural and surgical care, and communicating with patients and their family members.

In this evaluation we assessed the impact of the Student Assistantship Program on the first cohort of graduates using quantitative and qualitative data collected from both students and their clinical supervisors in their Post Graduate Year 1 posts. Our research questions were: In advance of the Student Assistantship Program, how prepared did our students feel to begin their roles as junior doctors? What impact did the Student Assistantship Program have on their sense of preparedness and experience of acute care situations? What changes to SAP would our graduates and their PGY1 supervisors suggest for the future to enhance its value for future cohorts of students?

## Methods

### Participants

Members of 2018 MBBS cohort of the Lee Kong Chian School of Medicine and their clinical supervisors in their first junior doctor posting.

### Ethics and consent

Information sheets detailing the studies aims and objectives were provided, and written consent was obtained from all participants. The study was approved by the Nanyang Technological University Institutional Review Board (IRB ID: IRB-2018–01-015) and National Healthcare Group Domain Specific Review Board Approval (NHG DSRB Ref: 2018/00490).

### Data collection from students

All medical graduates were invited at two time points (just before the start of SAP and immediately after SAP to complete an online questionnaire. We used an existing questionnaire developed to assess readiness for practice in the UK [13] which was based on the activities, standards and experiences required of doctors by the UK General Medical Council [14]. With the author’s permission we discussed the relevance of its contents with local clinicians and four adaptations were made to content to better reflect local common ‘acute care situations’: opiate overdose (OD) was replaced with paracetamol OD to reflect the most prevalent cause of poisoning [15]; stroke was added as it ranks third in disease burden, accounting for 6.8% of disability-adjusted life-years [16]; adverse drug reactions were added being responsible for 8.1% hospital admissions [17]; and anaphylaxis was removed as it was perceived as a rarely encountered clinical problem. Further detail about the questionnaire is summarised in (Table 1) were administered online using the Qualtrics survey platform (https://www.qualtrics.com).

**Table 1:** Details of Questionnaire used to evaluate impact of SAP on students

The questionnaire asked about students’ levels of preparedness, opportunities and experience. It was used with the original authors’ permission^10^ with minor modification to content to better reflect local common ‘acute care situations’ in Singapore
*Levels of preparedness* for clinical tasks was assessed by asking respondents to *“Please indicate how prepared you are to begin PGY1 in the following areas?”* This was followed by 37 capabilities. For example, “Formulate a plan for treatment, management and discharge” (question #7), Perform and interpret a 12-lead electrocardiograph (question #24), Prescribe fluids for intravenous infusion and set up infusion device (question #33) Responses were on a 5-point Likert-like scale (anchored “not at all prepared” (scoring 1) to “fully prepared”(5).
*Opportunities for practice during clinical placements* were assessed by four items. One was a yes/no response to a statement “*During my final year I had at least one attachment where I assisted a junior doctor undertaking most of the duties of a Foundation Year one doctor”*. This was followed by three statements about opportunities in relation to supervised clinical practice; *“I had the opportunity to make prescribing recommendations for the prescription of drugs”, “I had the opportunity carry out common procedures on patients under supervision” and “I had the opportunity to manage acutely unwell patients under supervision.”* Responses were rated on a 5-point Likert-like scale anchored from 1 ‘completely disagree’ to 5 ‘completely agree’
*Opportunities for practice during clinical placements* were assessed by four items. One was a yes/no response to a statement “*During my final year I had at least one attachment where I assisted a junior doctor undertaking most of the duties of a Foundation Year one doctor”*. This was followed by three statements about opportunities in relation to supervised clinical practice; *“I had the opportunity to make prescribing recommendations for the prescription of drugs”, “I had the opportunity carry out common procedures on patients under supervision” and “I had the opportunity to manage acutely unwell patients under supervision.”* Responses were rated on a 5-point Likert-like scale anchored from 1 ‘completely disagree’ to 5 ‘completely agree’
*Experience of acute care situations and readiness to be the first respondent during clinical placements,* were ascertained by asking *“In your final year have you experienced any of the following acute care situations in real life and what was your involvement?”* followed by ten common emergency situations (acute coronary syndrome, asthma, COPD exacerbation, diabetic ketoacidosis, GI bleed, paracetamol overdose, pulmonary embolism, sepsis, stroke and adverse drug reaction). Respondents indicated their level of involvement as “Lead”, “Participant”, “Observer” or “Not seen”. Leading was defined as “assessing the patient, starting initial management, and seeking help as appropriate” and participation as having some “hands on role e.g. placing a canula, airway manoeuvres etc.”. Relating to these ten acute care situations respondents were asked, *“Based on your experience in Final year, how ready do you feel to be the first respondent in the following acute care situations?”* The 5-point Likert-like scale responses ranged from ‘not at all ready’ (scoring 1) to ‘very ready’ (scoring 5)

To get a clearer understanding of the characteristics of SAP that the students valued we asked for volunteers to participate in two one-to-one semi-structured interviews about their experiences of SAP and how it impacted on their preparedness as a PGY1. This convenience sample were interviewed during the second SAP posting (weeks 4 to 7) and again, one month after starting their PGY1 posting. The one-on-one interviews with the students were conducted by three researchers (two female, one male) experienced in qualitative research methods. The interviewers were not known by the students and none of them had contributed to the design or delivery of SAP. All interviewees were informed about the research objectives.

### Data collection from PGY1 supervisors

Using a short questionnaire, with closed and open questions, the PGY1 clinical supervisors were asked to indicate their perceptions of how well prepared our graduates were when working as PGY1 doctors with respect to their skills, knowledge, attitudes, and professionalism, dealing with acute situations and knowing when to call for help. These characteristics were measured on a 6-point scale from ‘not prepared at all’ [1] to fully prepared [6]. On a 5-point scale they indicated their assessment of the effectiveness of SAP and responded ‘yes’ or ‘no’ to the statement ‘If your family member needed medical attention would you be happy for them to be part of the medical team caring for them?’. Open-ended questions asked about areas in which the PGY1s were well prepared and those where preparation could be improved. The link to the questionnaire was sent to PGY1 supervisors via email and administered online using the Qualtrics survey platform. Some PGY1 clinical supervisors also agree to participate in a one-to-one semi-structured interview to enable more in-depth exploration of the benefits of SAP and the attributes and weaknesses of graduates.

### Data analysis

The quantitative data of the students’ levels of preparedness were analysed using descriptive statistics generated using SPSS premium version 25 [18]. As the questionnaire used a Likert-like scale, with numbers rather than categories we used parametric analyses [19, 20]. The second data set had 6% randomly missing data, so we used Rubin’s method of multiple imputation for non-response to generate 10 imputed datasets, and pooled the results [21, 22].

### Interview analysis

Interviews were audio-recorded, transcribed verbatim and analysed thematically by the interviewers and principal investigator (BB, SC, AC and HES), They coded a sample of transcripts independently, before meeting to discuss and agree a coding framework that was then applied to all transcripts [23]; a similar process was repeated for the supervisors’ transcripts. The qualitative findings are reported using the Consolidated Criteria for reporting Qualitative Research (COREC) [24].

## Results

Fifty (96%) students responded to the questionnaire before SAP and 46 (92%) on completion of SAP. They ranged in age from 23 to 27 years old and 68% were male.

### Preparedness

Pre-SAP the modal scores for the 37 items of clinical preparedness ranged from 2 to 5 on a scale where 1 represented ‘not at all prepared’ and 5 ‘fully prepared’, with mean scores ranging from 2.38 to 4.32 (Table 2). Post-SAP the levels of preparedness were generally higher (scores ranged from 3 to 5; mean scores from 3.08 to 4.48). All but two of the 37 items of preparedness showed an upward trend post-SAP, 20 improvements were statistically significant. (Table 2) Overall scores are a good indicator of the cohort’s readiness, but may mask individual’s suboptimal performance. Pre-SAP there were 16 clinical practice areas where $\ge$ 10% students self-assessed themselves as unprepared, with scores of 1 or 2. Unpreparedness ranged across different types of skills, including prescribing, formulating plans, communicating and procedures. “Prescribe, set up and monitor a blood transfusion” had the least preparedness (56% scoring 1 or 2) and post-SAP the proportion unprepared decreased to 17%. (Table 3) The three other areas where unpreparedness remained ≥ 10% were “Contribute to the care of patients and their families at the end of life” (15%), “Perform a mental-state examination” (24%), and “Carry out basic respiratory function tests” (17%) (Table 3).

**Table 2:** Comparisons of student’s scores of preparedness for clinical practice before and after SAP (*N* = 50)

Levels of Preparedness in Clinical Practice	Scores before SAP		Scores on completion SAP	
	**Mean (SE)**	**Mode**	**Mean (SE)**	**Mode**
Take and record a patient's medical history, including family and social history?	3.96 (.565)	4	4.27* (.439)	4
Elicit patients' questions, their understanding of their condition and treatment options, and their views, concerns, values and preferences?	3.88 (.712)	4	4.18* (.572)	4
Perform a full physical examination?	3.92 (.659)	4	4.23* (.602)	4
Perform a mental-state examination?	3.02 (.788)	3	3.20 (1.130)	4
Make an initial assessment of a patient's problems and formulate a differential diagnosis?	3.58 (.724)	4	3.98* (.503)	4
Formulate a plan for investigation and interpret the results?	3.54 (.781)	4	4.00* (.475)	4
Formulate a plan for treatment, management and discharge?	3.06 (.733)	3	3.61* (.615)	4
Make clinical judgements and decisions, based on the available evidence, in conjunction with colleagues?	3.22 (.783)	3	3.82* (.513)	4
Contribute to the care of patients and their families at the end of life?	2.84 (.903)	3	3.24* (.786)	3
Communicate effectively with colleagues from a variety of professions?	3.38 (.690)	3	3.80* (.577)	4
Communicate clearly, sensitively, and effectively with patients, relatives or other carers?	3.40 (.775)	3	3.94* (.640)	4
Communicate appropriately in difficult circumstances (e.g. with difficult or violent patients, when breaking bad news, or with vulnerable patients?	3.00 (.722)	3	3.46* (.821)	3
Assess and recognise the severity of a clinical presentation and a need for immediate emergency care?	3.58 (.778)	4	3.93* (.688)	4
Diagnose and manage acute medical emergencies?	3.52 (.831)	4	3.63 (.774)	4
Provide cardio-pulmonary resuscitation or direct other team members to carry out resuscitation?	3.68 (.787)	4	3.62 (.980)	4
Plan appropriate drug therapy for common indications, including pain and distress?	3.10 (.756)	3	3.72* (.549)	4
Provide a safe and legal prescription?	2.86 (.694)	3	3.61* (.564)	4
Calculate appropriate drug doses?	2.96 (.872)	3	3.51* (.720)	3^a^
Detect and report adverse drug reactions?	3.34 (.682)	3	3.45 (.627)	3
Keep accurate, legible and complete clinical records?	3.38 (.719)	3	4.05* (.600)	4
Carry out baseline observations: measuring body temperature, pulse rate, blood pressure, transcutaneous oxygen monitoring (saturation monitoring)?	4.32 (.647)	4	4.48 (.612)	5
Carry out practical procedures: venepuncture, taking blood cultures, measuring blood glucose?	4.12 (.739)	4	4.27 (.655)	4
Establish peripheral intravenous access (cannulation)?	3.96 (.721)	4	4.02 (.740)	4
Perform and interpret a 12-lead electrocardiograph?	4.18 (.623)	4	4.08 (.687)	4
Carry out basic respiratory function tests?	3.16 (1.028)	3	3.18 (1.054)	3
Carry out practical procedures: urine multi dipstick test, taking nose, throat and skin swabs, pregnancy test?	3.60 (.693)	4	3.66 (.788)	3
Administer oxygen?	4.14 (.749)	4	4.17 (.605)	4
Prescribe dose and route of insulin, including use of sliding scales?	3.60 (.849)	4	3.83 (.648)	4
Administer subcutaneous and intramuscular injections?	3.92 (.797)	4	4.01 (.805)	4
Carry out practical procedures: urinary catheterisation, skin suturing?	3.90 (.782)	4	4.09 (.692)	4
Wound care and basic wound dressing?	3.60 (.775)	4	3.76 (.769)	4
Infection control (e.g. hand washing, use of personal protective equipment, safe disposal of waste and sharps)?	4.32 (.734)	5	4.39 (.642)	5
Prescribe fluids for intravenous infusion and set up infusion device?	3.38 (.719)	3	3.66* (.705)	4
Prescribe, set up and monitor a blood transfusion?	2.38 (.978)	2	3.08* (.721)	3
Hand over care of a patient?	2.96 (.721)	3	3.72* (.548)	4
Know when to seek help from a senior colleague?	3.92 (.689)	4	4.07 (.541)	4
Learn and work effectively within a multi-professional team?	3.44 (.753)	3^a^	4.09* (.513)	4

^a^multiple modes exist, this is the lowest value

^*^dependent t-test was statistically significant at *p* < .05

**Table 3:** The sixteen activities for which 10% or more of respondents felt unprepared pre-SAP, together with the proportion post-SAP

Activity	Time Points	% Unprepared (No.)	1 *Not at all prepared*	2	3	4	5 *Fully prepared*
Prescribe, set up and monitor a blood transfusion	Pre SAP (*n* = 50)	56.0 (28)	14%	42%	30%	10%	2%
	Post SAP (*n* = 46)	17.4 (8)	2%	14%	50%	26%	0%
Contribute to the care of patients and their families at the end of life	Pre SAP	32.0 (16)	2%	30%	44%	20%	2%
	Post SAP	15.2 (7)	2%	12%	42%	34%	2%
Calculate appropriate drug doses	Pre SAP	30.0 (15)	4%	26%	42%	26%	2%
	Post SAP	6.5 (3)	0%	6%	40%	40%	6%
Provide a safe and legal prescription	Pre SAP	28.0 (14)	2%	26%	56%	16%	0%
	Post SAP	4.3 (2)	0%	4%	28%	60%	0%
Communicate appropriately in difficult circumstances (*e.g. with difficult or violent patients, when breaking bad news, or with vulnerable patients*)	Pre SAP	24.0 (12)	0%	24%	54%	20%	2%
	Post SAP	8.7 (4)	2%	6%	42%	34%	8%
Hand over care of a patient	Pre SAP	24.0 (12)	2%	22%	54%	22%	0%
	Post SAP	0.0 (0)	0%	0%	30%	58%	40%
Perform a mental-state examination	Pre SAP	22.0 (11)	2%	20%	56%	18%	4%
	Post SAP	23.9 (11)	4%	18%	26%	32%	10%
Carry out basic respiratory function tests	Pre SAP	22.0 (11)	2%	20%	38%	30%	8%
	Post SAP	17.4 (8)	0%	16%	36%	30%	6%
Make clinical judgements and decisions, based on the available evidence, in conjunction with colleagues	Pre SAP	20.0 (10)	0%	20%	40%	38%	2%
	Post SAP	0.0 (0)	0%	0%	22%	66%	4%
Formulate a plan for treatment, management and discharge	Pre SAP	18.0 (9)	2%	16%	58%	22%	2%
	Post SAP	2.2 (1)	0%	2%	34%	52%	4%
Plan appropriate drug therapy for common indications, including pain and distress	Pre SAP	18.0 (9)	2%	16%	54%	26%	2%
	Post SAP	2.2 (1)	0%	2%	24%	64%	2%
Formulate a plan for investigation and interpret the result	Pre SAP	10.0 (5)	2%	8%	28%	58%	4%
	Post SAP	0.0 (0)	0%	0 (0%	10%	72%	10%
Communicate clearly, sensitively, and effectively with patients, relatives or other carers	Pre SAP	10.0 (5)	0%	10%	48%	34%	8%
	Post SAP	0.0 (0)	0%	0%	22%	54%	16%
Detect and report adverse drug reactions	Pre SAP	10.0 (5)	0%	10%	48%	40%	2%
	Post SAP	4.3 (2)	0%	4%	44%	42%	2%
Prescribe dose and route of insulin, including use of sliding scales	Pre SAP	10.0 (5)	0%	10%	34%	42%	14%
	Post SAP	2.2 (1)	0%	2%	22%	58%	10%
Learn and work effectively within a multi-professional team	Pre SAP	10.0 (5)	0%	10%	42%	42%	6%
	Post SAP	0.0 (0)	0%	0%	8%	68%	16%

Footnote: The activities are ordered by degree of initial unpreparedness, from the highest to the lowest

### Experience

Post-SAP the proportion of students with opportunities to “assist a junior doctor to undertake most of the duties of a Foundation Year 1 doctor” had increased from 56% (28/50) to 96% (46/50). SAP provided increased opportunities in all three areas explored: making recommendations for drug prescribing (mean score increased from 2.68 to 4.15, *p* < 0.001), manage acutely unwell patients (2.60 to 3.78, *p* < 0.001), and to carry out common procedures (3.64 to 4.39, *p* < 0.001) (Table 4). SAP also enhanced hands-on experience of providing immediate care in a medical emergency, from 76% pre-SAP to 89% post-SAP.

**Table 4:** Frequencies of responses to questions about opportunities in clinical practice

Opportunities in Clinical Practice	Time Points	Mean score (SE)^a^	*p*-value	Mode score^a^	Frequencies (%)				
					**1 *Completely disagree***	**2**	**3**	**4**	**5 *Completely agree***
I had the opportunity to make recommendations for the prescription of drugs	Pre SAP (*n* = 50)	2.68 (.969)	*p* < .001	3	6 (12%)	15 (30%)	19 (38%)	9 (18%)	1 (2%)
	Post SAP (*n* = 46)	4.16 (.658)		4	0 (0%)	1 (2%)	4 (9%)	28 (61%)	13 (28%)
I had the opportunity to carry out common procedures on patients under supervision	Pre SAP	3.64 (1.092)	*p* < .001	4	3 (6%)	4 (8%)	12 (24%)	20 (40%)	11 (22%)
	Post SAP	4.38 (.643)		5	0 (0%)	0 (0%)	4 (9%)	20 (43%)	22 (48%)
I had the opportunity to manage acutely unwell patients under supervision	Pre SAP	2.60 (1.150)	*p* < .001	2	10 (20%)	15 (30%)	12 (24%)	11 (22%)	2 (4%)
	Post SAP	3.78 (.859)		4	1 (2%)	3 (7%)	8 (17%)	27 (59%)	7 (15%)

^a^based on multiple imputation analysis

Real life experience of acute care situations increased significantly for asthma, chronic obstructive pulmonary disease (COPD) exacerbation, diabetic ketoacidosis, gastrointestinal (GI) bleed, pulmonary embolism, sepsis, stroke, and adverse drug reaction but not for paracetamol overdose (Table 5). The mean scores for readiness to be first respondent in such acute situations improved for all ten, but the differences were only statistically different for asthma, COPD, GI bleed, sepsis, and adverse drug reactions (Table 6).

**Table 5:** Comparisons of experience of real-life acute care situations for students pre- and post-SAP

Questionnaire item	Time Points	Mean score (SE)^a^	Modal response^a^	Frequencies (% of completed responses)				
				**Lead**	**Participant**	**Observer**	**Not seen**	***Missing data***
In your final year, have you had experience of any of the following acute care situations in real life?								
Acute coronary syndrome	Pre SAP (*n* = 50)	1.20 (.954)	Participant	5%	41%	22%	32%	9
	Post SAP (*n* = 46)	1.43 (.150)	Participant	12%	44%	20%	24%	5
Asthma	Post SAP	1.33 (.879)	Participant	4%	47%	27%	22%	5
	Pre SAP	1.76* (.151)	Participant	19%	55%	10%	17%	4
Chronic obstructive pulmonary disease exacerbation	Post SAP	1.29 (.835)	Participant	2%	45%	31%	21%	8
	Pre SAP	1.73* (.148)	Participant	19%	56%	7%	19%	3
Diabetic ketoacidosis	Post SAP	1.05 (.893)	Observer	5%	27%	37%	32%	9
	Pre SAP	1.47* (.151)	Participant	12%	51%	9%	28%	3
Gastrointestinal bleed	Post SAP	1.05 (.854)	Observer	2%	31%	36%	31%	8
	Pre SAP	1.88* (.111)	Participant	12%	71%	10%	7%	4
Paracetamol overdose	Post SAP	.59 (.785)	Not seen	0%	18%	23%	59%	11
	Pre SAP	.50 (.109)	Not seen	0%	16%	14%	70%	9
Pulmonary embolism	Post SAP	.83 (.813)	Not seen	0%	25%	33%	43%	10
	Pre SAP	1.27* (.148)	Participant	8%	41%	21%	31%	7
Sepsis	Post SAP	1.26 (.902)	Participant	2%	49%	21%	28%	7
	Pre SAP	2.17* (.103)	Participant	33%	53%	11%	2%	1
Stroke	Post SAP	1.15 (.882)	Participant	2%	39%	29%	29%	9
	Pre SAP	1.77* (.140)	Participant	21%	53%	37%	19%	3
Adverse drug reaction	Post SAP	.61 (.679)	Not seen	0%	11%	39%	50%	12
	Pre SAP	1.04* (.167)	Not seen	8%	32%	16%	45%	8

^a^based on multiple imputation analysis

^*^dependent t-test was statistically significant at *p* < .05

**Table 6:** Comparisons of scores before and after SAP relating to readiness to be first respondent in acute care situations (*n* = 50)

How ready do you feel to be the first respondent in the following acute care situations? *(First respondent means that you are able to perform initial assessment and management before the arrival of any of your senior team)*	**Time Points**	**Mean score (SE)**^a^	**Mode score**^a^	**Frequencies (%)**					
				**0 *Don’t know***	**1 *Not at all ready***	**2**	**3**	**4**	**5 *Very ready***
Acute coronary syndrome	Pre SAP	3.46 (.964)	4	1 (2%)	0 (0%)	7 (14%)	13 (26%)	25 (50%)	4 (8%)
	Post SAP	3.65 (.782)	4	0 (0%)	0 (0%)	5 (11%)	10 (22%)	27 (59%)	4 (9%)
Asthma	Pre SAP	3.36 (.955)	4	1 (0%)	0 (0%)	9 (18%)	12 (24%)	26 (52%)	2 (4%)
	Post SAP	3.74* (.669)	4	0 (0%)	0 (0%)	2 (4%)	12 (26%)	28 (61%)	4 (9%)
Chronic obstructive pulmonary disease exacerbation	Pre SAP	3.26 (.977)	4	1 (2%)	0 (0%)	10 (20%)	16 (32%)	20 (40%)	3 (6%)
	Post SAP	3.70* (.695)	4	0 (0%)	0 (0%)	2 (4%)	13 (28%)	27 (59%)	4 (9%)
Diabetic ketoacidosis	Pre SAP	3.52 (.986)	4	1 (2%)	0 (0%)	7 (14%)	11 (22%)	26 (52%)	5 (10%)
	Post SAP	3.78 (.632)	4	0 (0%)	0 (0%)	1 (2%)	12 (26%)	29 (63%)	4 (9%)
Gastrointestinal bleed	Pre SAP	3.30 (.944)	4	1 (2%)	0 (0%)	8 (16%)	18 (36%)	20 (40%)	3 (6%)
	Post SAP	3.74* (.673)	4	0 (0%)	0 (0%)	2 (4%)	12 (26%)	28 (61%)	4 (9%)
Paracetamol overdose	Pre SAP	2.72 (.873)	2^b^	1 (2%)	1 (2%)	19 (38%)	19 (38%)	10 (20%)	0 (0%)
	Post SAP	2.84 (.929)	3	1 (2%)	2 (4%)	13 (28%)	18 (39%)	12 (26%)	0 (0%)
Pulmonary embolism	Pre SAP	3.20 (1.001)	3^b^	1 (2%)	1 (2%)	9 (18%)	18 (36%)	18 (36%)	3 (6%)
	Post SAP	3.36 (.787)	4	0 (0%)	0 (0%)	8 (17%)	15 (33%)	22 (48%)	1 (2%)
Sepsis	Pre SAP	3.44 (.899)	4	1 (2%)	0 (0%)	6 (12%)	14 (28%)	27 (54%)	2 (4%)
	Post SAP	3.83* (.634)	4	0 (0%)	0 (0%)	1 (2%)	10 (22%)	30 (65%)	5 (11%)
Stroke	Pre SAP	3.20 (.873)	3	1 (2%)	0 (0%)	8 (16%)	21 (42%)	19 (38%)	1 (2%)
	Post SAP	3.42 (.809)	4	0 (0%)	1 (2%)	4 (9%)	17 (37%)	22 (48%)	2 (4%)
Adverse drug reaction	Pre SAP	2.74 (.891)	3	1 (2%)	1 (2%)	18 (36%)	21 (42%)	8 (16%)	1 (2%)
	Post SAP	3.10* (.849)	3	0 (0%)	1 (2%)	10 (22%)	19 (41%)	15 (33%)	1 (2%)

^a^based on multiple imputation analysis

^b^multiple modes exist, this is the smallest value

^*^dependent t-test was statistically significant at *p* < .05

#### Student interviews

During SAP 16 students were interviewed and 11 re-interviewed on completion of their first four-month PGY1 attachment. Three major themes were constructed: Learning about the work environment, Opportunities to learn in a safe environment and Enhancing SAP for future students. These themes are illustrated below with quotes and unique identifiers representing the participant’s study number (two digits), and a second number indicating if the interview was conducted during SAP [1] or in PGY1 [2].

### Learning about the work environment

Students perceived SAP as complementing their undergraduate clinical rotations, providing hands-on experience and contextual knowledge of the work environment, which they perceived as essential for the practice of medicine:

“*…it’s [SAP] also not only about applying knowledge you learn in medical school, but also learning how the system works here, because without the system there’s no medicine.*” (01-1)

*“Some kind of trial run, you know, um, some kind of simulation, test drive, before you go into the real world.”* (20-2)

SAP was experienced as a bridge, facilitating the transition from student to healthcare professional:

*“I think in general when you’re given responsibility to see patients it strengthens your identity as a doctor, which is quite a big thing before we step out into actual practice, because you have to lose that identity as a medical student, ….and start thinking professionally and realizing that people are looking at you as a professional.” (03-1)*

*“Meeting the other HOs and learning from them and their experiences, giving good advice about how it was like when they first started being a doctor, and the first weeks when they start being a doctor…knowing about their experiences makes me feel a bit more comforted, it sometimes makes me feel a little more confident.” (40-1)*

*“So, I think we are basically working at the level of junior HO and I can see a patient and then… write the notes, write the documentation of the patient as well. And afterward discuss the plans with a senior, like a medical officer or a HO, what to do.” (09-1)*

For some students, this period of transition generated by SAP acted as a wakeup call, emphasizing the responsibilities and stresses of being a PGY1.

*“During SAP I suddenly had a turnaround, because it kind of dawned on me… soon I am going to be the first line respondent to a lot of these situations, and a lot of responsibility is going to fall on me… playtime is over. It’s time to buck up and get serious.” (20-2)*

This insight into the realities of life as a junior doctor reinforced the need for being emotionally prepared for a challenging work environment that contrasts markedly with the support available in medical school:

*“During SAP also realised that PGY1 or HO year is definitely a tough year. The working environment can be very hostile sometimes. Yes, and I must be prepared for that when I start work …. But some PGY1s who are very burnt out, very unhappy with work life …. it’s good to see it at the start, before you start. So that when you start you can make a conscious decision to stay away from these things and to prevent ourselves from becoming like that.” (39-2)*

#### Opportunities to learn in a safe environment

SAP was considered a safe context in which to learn, representing a *“half-way house”* with respect to both workload and work responsibilities. Many respondents spoke about SAP enabling them to build their confidence and efficiency in a protected environment:

*“… we get to take on a small proportion of the duties that the house officer takes on. This scope is similar, but the amount is less because they don’t expect us to … take charge of all the patients that a team has… As by doing things incrementally in this way I feel that it allows us to better ease into the duties that we have in the future.” (19-1)*

*“So, it’s like being a doctor with safety netting. I always have a doctor with me to check on my work and make sure that I’m doing something right, and to tell me when I’m not.” (20–1)*

*“I think the SAP is good for us to make mistakes because we are still not accountable yet. It’s not medically liable for any, any mistakes that you might make. As opposed to when you are making mistakes as a house officer.” (26-1)*

#### Students’ suggestions for improving SAP

The SAP experiences recalled spontaneously were all positive, and only when prompted did graduates offer suggestions for improvement. These included:

1. Reviewing the utility of polyclinic placements in SAP
  *“…the polyclinic postings we do for SAP might be a step too high for us. Because they are usually medical officer postings, and not like year five postings, so we don’t really get enough experience and enough exposure to handle cases on our own.” (45-1)*
2. Making SAP more realistic, with greater accountability for decisions made
  *“It’s not entirely realistic because …. they still treat you as student, so you are shielded from the complicated parts- it’s very different working and doing SAP.” (01–2)*

*“…. you can’t be sheltered all the time, but you need to be given responsibility, so you know what it is to have responsibility and what to do with your responsibility. So, it won’t be so much like a sheltered experience but more like a real-life working experience. Your good times, bad times you need to know how to handle, even as a student.” (01-2)*

3) Organising placements in more hospitals
  “*I think we should expose everyone to more hospitals, at least maybe three hospitals, you can’t do your postings in the same hospital twice.” (01-2)*
4) Standardisation of attachments
  *“Teams were struggling with manpower, even when we come in we are kind of expected to function as junior doctors, but I think lots of us still don’t know the intricacies of the computer systems… there’s very little time allocated to teaching. I think the junior doctors sometimes felt that, oh you know, there’s no point teaching you because learning is when you come out to work, or rather like I can teach you, but it will take five minutes, whereas I can just do it in one minute. Yeah, so they would rather do it themselves.” (35-1)*
  Clinical teams’ ability and willingness to accommodate the SAP students varied. There was a sense that in the busy specialities, such as general surgery*:*
5) More guidance on how to behave in the workplace

SAP participants’ described difficulties coping with stress, being *“scolded for mistakes”* and difficulty supporting and communicating effectively with their seniors. Some generic guidance on coping was requested, together with formal scheduling of meeting for debriefing and support:

“I realise that when we actually started work and when we start SAP we see things they may not be ideal…. How do I deal with certain situations? How this, maybe have been better addressed in SAP.” (39–1)

“So, may be able to talk about that [stress in SAP], because that’s a challenge that we will face when working in the future.” (05–1)

### Supervisor’s perspectives

Supervisors from four hospitals completed the questionnaire (*n* = 33). 70% supervisors judged SAP as ‘very’ or ‘extremely effective’ for preparing students to become PGY1 doctors. The preparedness of LKCMedicine graduates for practice as PGY1 doctors was assessed as more than 4.5/6 for all characteristics (Table 7), with Attitudes and Professionalism scoring highest (4.9) and dealing with acute care situations the least at 4.5. 97% responded positively when asked ‘If your family needed medical attention would you be happy for them to be part of the team?” Eight supervisors (S1-8) agreed to be interviewed to provide more detailed feedback.

**Table 7:** Supervisors assessment of the preparedness for practice as a PGY1 doctor (*N* = 33)

How well prepared are LKCMedicine graduates for practice as PGY1 doctors in the following areas?	Mean	Mode	1 *Not at all prepared*	2	3	4	5	6 *Fully prepared*
Skills	4.7	5	0 (0%)	0 (0%)	1 (3%)	10 (30%)	19 (58%)	3 (9%)
Knowledge	4.8	5	0 (0%)	1 (3%)	1 (3%)	8 (24%)	17 (52%)	6 (18%)
Attitudes and professional behaviour	4.9	5	0 (0%)	0 (0%)	1 (3%)	8 (24%)	17 (52%)	7 (21%)
Dealing with acute care situations	4.5	4	0 (0%)	0 (0%)	2 (6%)	15 (45%)	14 (42%)	2 (6%)
Knowing when to call for help	4.7	4	0 (0%)	0 (0%)	0 (0%)	14 (42%)	14 (42%)	5 (15%)

### Attributes of SAP

Supervisors spoke positively about the content, timing, and duration of SAP

*‘Three postings they do. And those three postings are enough, the time duration is enough to build up the resilience, to build up the attitude, skills and to make them prepare for PGY1*’ (S4)

They saw SAP distinct from any of the preceding clinical rotations:

*“So effectively they devote all their attention and all their capabilities and abilities to learn clinical work. Manage patients, do hands on stuff”* (S4)

The impact of SAP was felt to be enhanced by its positioning after final exams, enabling the students to give their full attention to preparing for work:

*“So they are, they’re not so much worried about trying to find cases to examine, to plot, to prepare for their exams, but rather their actual internship itself” (*S1)

Hosting assistantships was recognised as a team effort, requiring engagement of all the team, ‘*the consultant, the registrar, the medical officer, the current resident, um, will be involved in the day to day running of the SAP program’* (S1). The variations in team structure and differences in workloads, meant there was an element of *‘luck’* in SAP placements and supervisors spoke about the need to minimise variation with supervisor training and feedback to each team, blacklisting those who provide little involvement of the students.

### Refinements to SAP

1. Inclusion of activities to development of coping skills

SAP was recognised as offering a relatively sheltered environment in which to help students prepare emotionally for PGY1.

‘*It is a supported environment. So, they feel if ‘I don't know anything about it I can ask somebody’, and they know whom to ask. So automatically that gives them that buffer around them and ‘I am not alone,… if I make a mistake and I don't know anything there is somebody to cover me*’ (S4)

In this protected context, it was suggested students could be taught ways of coping with diffcult situations they will encounter, such as “bad outcomes or patients who are not so nice because at some point of time, all PGY1s will encounter, like difficult relatives, you know, difficult patients” (S2).

2) Establishing a habit of reflection

Another suggestion was the development of reflective skills, and incorporation of protected time within SAP for the students to reflect on their actions:

“*As a daily exercise, or maybe weekly exercise. Because this part is something that could be lost when a doctor is very, very busy, and then they just go through the motions. And without knowing it… a few months has passed and then they are onto another posting. So, I think this part about reflecting and, and thinking about what do these reflections or what lessons can they draw from these reflections to apply to the next patient*” (S2)

Some refinements proposed were desirable but recognised as difficult to organize, for example, providing SAP placements in the same hospitals as the PGY1 postings, to avoid *‘having to re-learn the system’ (S8).* or providing more opportunities for practical procedures when SAP students are in direct competition with PGY1s for such learning opportunities, and the latter have priority, “W*ill let the PGY1 gain more competency first.”* (S6).

## Discussion

### Summary of findings

This before and after study demonstrated that the Student Assistantship Program was successful increasing levels of preparedness and experience across a wide range of clinical areas, and frequently these improvements were statistically significant. Students perceived the program as a bridge between undergraduate curriculum and work; their attachments being conducted in a safe environment created by a reduced workload and supervision. The SAP provided valuable opportunities for graduates to hone their skills and to learn from junior doctors. The students recognised variation between SAP placements, some having less immediate relevance to PGY1 (e.g., primary care placements in polyclinics), and those where the team’s routine heavy caseload reduced opportunities for accommodating students gaining hands-on experiences. In considering SAP for the future students wondered if their experience could be more realistic with them having greater accountability for the decisions they made. They also suggested greater guidance on professional behaviour, including responding to reprimands and communicating with their superiors. Most supervisors judged SAP as very or extremely effective preparing students to become PGY1 doctors and with the exception of one supervisor they were willing for our graduates to be part of the team attending to one of their family. The supervisor’s suggestions for SAP development were often congruent with the students’ ideas. For example, they too recognised a need to standardise placements through training of and feedback to supervisors, blacklisting teams who were unable to provide sufficient student involvement. They identified a need to enhance students’ coping skills. Supervisors saw this as a need to cope with difficult patients or relatives, whereas the students expressed their need more in the context of coping with colleagues. Some supervisors suggested that SAP could be an ideal opportunity to introduce the students to reflection, with protected time set aside within SAP for them to think about the lessons learnt. This skill could become an integral part of the clinical practice and assist in their lifetime learning.

None of the students or supervisors suggested any changes should be made to the structure of the SAP module. However, comparisons of students in a Longitudinal Integrated Clerkships (LICs) rather than traditional block rotations appear to engage more actively in patient care, functioning in a doctor-like role. [25, 26] Changing SAP from a rotation between three placements, to a single, more integrated and longitudinal placement may enhance SAP’s impact, but the LIC model is still in its infancy in Asia [27, 28]. Even in regions where LICs are more established students describe significant challenges transitioning between the two clerkship models [29]. It could be argued that it may not be appropriate for us to impose a transition in learning style at the same time as the students grapple with transitioning from student to doctor.

### Strengths and weaknesses

This rigorous longitudinal mixed methods evaluation adds to the previously sparce data on the efficacy of assistantships. A strength of this formal evaluation confirming the educational value of this SAP module is that it is a before-and-after study rather than a simple cross-sectional study. The evidence of SAP success is strengthened by the inclusion of feedback from their PGY1 supervisors at the start of the junior hospital posts. We incorporated the supervisor perspective because we recognised the potential problems of basing assessment of preparedness on student’s self-assessment of ‘preparedness’, when there may be variation in respondents understanding of the concept and their responses may be contaminated by anxiety or confidence. Having the perspective of clinical supervisors, who have experience of working with medical students from other schools, observing our graduates as they start their junior doctor placements, brings a comparative and more independent assessment of preparedness to our findings.

By using mixed methods our findings have added value; the quantitative data helps understand the characteristics of readiness within the student population whilst the qualitative data highlights potential refinement of the program’s content and organisation that could enhance the quality and value of SAP in the future. These findings are early results from a single institution, the first cohort of graduates from a new medical school. Anecdotally it is said that the early entrants are unusual, as their undergraduate experience is marked by an absence of a senior peer group, and that the smaller cohort size means clinical placements are provided in a more limited range of healthcare institutions, and mentorship by medical school faculty is more personalised. This might limit the generalisability of our findings and we will repeat the evaluation in future years to confirm that the characteristics of being in the first cohort do not influence the impact of SAP.

### Comparison with other studies

Assistantship programmes are one of three types of transitional interventions widely used in preparation for junior hospital posts. The results of this study support the findings in the literature that transitional interventions can be efficacious in increasing the preparedness and confidence of medical graduates for their practice as doctors [5, 30, 31]. It is argued that medical graduates can never be totally prepared but helping them to be as prepared as possible will help mitigate harmful and serious consequences. To explore if SAP was able to impact on transforming the preparedness students when they rated poor preparedness, we conducted a before and after analysis for the 16 items where ≥ 10% students had scored in the lower half of the scale prior to SAP. This analytical approach had been used in a UK based study of doctors graduating from different medical schools (local and international) attending a foundation programme induction before starting jobs in the locality [10]. In that study there were seven areas of unpreparedness reported amongst doctors who were just about to start their house jobs (insulin dosing, blood transfusion, wound care, management of medical emergencies, adverse drugs reactions, practical procedures, care of patients and families at end of life). This data was collected at a time equivalent of our post-SAP data, but at this timepoint we observed only four areas where ≥ 10% were unprepared (blood transfusion, respiratory function testing, mental state examination, care of patients and families at end of life). This suggests that our graduates have equivalent or better preparedness than this heterogeneous sample of doctors who were about to commence work in the UK.

There are two influences on workplace learning, these are workplace affordances and individual engagement, and both are apparent in our data. The individual’s engagement with educational opportunities is apparent where students discuss their engagement being triggered by the pending responsibilities of patient management [32]. Even more prominent in our data are examples of affordance, that is those circumstances within the specific workplace and in workload which enable or prevent student engagement. Affordance examples included the heterogeneity of placements, the difficulty getting supervision when the team is busy, the difficulty to improve skills in mental health assessment in placements focusing on physical health disorders. One of the suggestions for improving SAP was allocating students to assistantships in the hospital and with the teams with which they would eventually work. There is evidence that in a non-aligned model the consultant may emotionally disinvest in students not allocated to work with then, knowing that they “will not reap the benefits” of the time invested. An aligned model would enhance the affordances available.

### Future research

The utility of SAP in future cohorts needs to be confirmed, ensuring that it benefits are sustained when the student cohort is larger. A bigger data set will also enable us to look for the characteristics of those students who derive less benefit from SAP and to formulate additional interventions to enhance their preparedness. Questions remain about the sustainability of the improvements we observed, and this can be addressed in part by the analysis of qualitative data collected at a third timepoint, after the students have started their the PGY1 placement. However, we recognise that longitudinal research spanning a transition can be very difficult, with attrition of participants as their contact details change and they become preoccupied with familiarising themselves with their new roles and responsibilities. We also wish to collect qualitative data over an extended period of time, rather than as a two-point follow-up study. Balmer et al. have advocated greater use of longitudinal qualitative research in medical education, as recursive data allows exploration of the student’s own journey *through time*, and where time is *fluid*. [33] Respondents can reflect on the past and how their perceptions change in the light of recent experience. It is suggested that the trust and safe space generated in recursive data collection may encourage rather than discourage retention of participants in the research. The actual observation of PGY1s within their clinical teams could also generate a more detailed understanding of the transition dynamics, but such studies must confine themselves to the observation of administrative tasks and interactions between healthcare professionals, as patient confidentiality requires the observer to withdraw when the junior doctor is interacting with patients. A previous study using observation techniques found that observation was more acceptable to experienced doctors, and most junior doctors declined to participate, perhaps because of their lack of confidence [34].

## In conclusion

In Singapore, there is no national requirement to have an assistantship, but all three medical schools do have one, each differing in content and timing. This is the first formal evaluation of an assistantship in Singapore, and the findings are encouraging from the perspective of students and clinical supervisors. This evaluation has focussed our attention on those areas of the five-year medical curriculum which need reviewing to enhance skill acquisition, and it has highlighted how the assistantship experience in the workplace can be modified to optimise students’ readiness to begin their first junior hospital post.

## Data Availability

The datasets used and analysed for this study are available from the corresponding author on reasonable request.

## Abbreviations

COPD: Chronic obstructive pulmonary disease
COREC: Consolidated Criteria for reporting Qualitative Research
GI: Gastrointestinal
LKCMedicine: Lee Kong Chian School of Medicine
OD: Opiate overdose
PGY1: Postgraduate Year 1
SAP: Student Assistantship Programme
WBA: Workplace-based assessments
